# Barriers to prophylactic treatment among patients with haemophilia A in Shandong Province, China: a qualitative study

**DOI:** 10.1186/s13023-023-02838-8

**Published:** 2023-08-03

**Authors:** Ziyu Liu, Junchao Feng, Yunhai Fang, Yan Cheng, Shunping Li

**Affiliations:** 1 Centre for Health Management and Policy Research, School of Public Health, Cheeloo College of Medicine, Shandong University, Jinan, 250012 China; 2 NHC Key Lab of Health Economics and Policy Research, Shandong University, Jinan, 250012 China; 3 Centre for Health Preference Research, Shandong University, Jinan, 250012 China; 4 Shandong Blood Center, Shandong Haemophilia Treatment Center, Jinan, China

**Keywords:** Haemophilia A, Prophylactic treatment, Barriers, Adherence, China

## Abstract

**Background:**

Haemophilia A is a rare, hereditary haemorrhagic disease that manifests as induced spontaneous bleeding and leads to disability or premature death in severe cases. Prophylactic treatment is optimal for patients to prevent uncontrolled bleeding and reduce the severity of the injury. However, little is known about the use of prophylactic treatment among patients with haemophilia A in China, especially barriers that predispose them to low or non-adherence. In this study, we explore the barriers to the prophylactic treatment of patients with haemophilia A.

**Method:**

We used personal interviews and focus groups to collect the data and analysed the data through thematic analysis. Purposive sampling was employed to recruit our participants. We continued recruiting participants until data saturation was reached from the thematic analysis. Ultimately, we obtained 37 participants, among whom 19 participated in personal interviews and 18 participated in focus groups (i.e., 3 focus groups with 6 participants each).

**Results:**

Three themes and nine subthemes were identified from the thematic analysis. Nine subthemes (i.e., perceived barriers) emerged from the analysis, which were further clustered into three themes: (1) poor primary health care, (2) inadequate financial support, and (3) a lack of patient-centred care.

**Conclusion:**

The findings presented in this descriptive qualitative study offer a unique view of Chinese patients with haemophilia A and their barriers to prophylactic treatment. Our findings not only provide an in-depth understanding of barriers to prophylactic treatment encountered by Chinese patients with haemophilia A but also address the urgent need to strengthen primary care, provide adequate financial support, and establish patient-centred care for these suffering patients.

## Introduction

Haemophilia A is a rare, X chromosome-linked bleeding disorder caused by partial or total deficiency of coagulation factor VIII (FVIII) [1]. As the most common subtype of haemophilia, haemophilia A represents 80–85% of the total haemophilia population [2]. The estimated prevalence at birth is 24.6 cases per 100 000 males for all severities of haemophilia A, but the prevalence is unclear in some countries, such as China [3]. In China, haemophilia continues to garner a great deal of attention from the Chinese government and was included in its first national list of rare diseases, given that it has a relatively large population of affected patients and poses a heavy burden to patients and their families [4, 5].

Unexpected bleeding after an injury is a common symptom in patients with haemophilia A [6]. In most cases, bleeding cannot stop on its own and often results in serious complications, such as joint bleeding and disabling haemophilic arthropathy [2, 7]. Replacing FVIII through on-demand infusion (episodic) or regular infusion (prophylactic) are the two treatment options available for patients with haemophilia A [8]. Compared with episodic treatment, prophylactic treatment demonstrates greater efficacy in reducing the incidence of joint haemorrhages and life-threatening haemorrhages, lowering the risk of joint damage, and improving the quality of life for patients [9, 10]. However, episodic treatment has continued to be most commonly used for patients who reside in less developed countries. Even for patients who initiate prophylactic treatment at an early stage, there has been significant evidence of a low level of adherence [11–14].

Previous studies have identified a wide range of perceived barriers and limitations to treatment adherence, including financial barriers [15], technical barriers related to medication (e.g., FVIII storage [16] and FVIII preparation [17]), infusion devices [17, 18], side effects of treatment (e.g., muscle pain and psychological-related discomfort caused by infusion) [19], and poor health literacy of patients [20]. Although these barriers have been explored and synthesized within a wide range of contexts, little attention has been given to assessing the prophylactic treatment adherence of patients living in China.

This study aimed to explore barriers to prophylactic treatment among patients with haemophilia A and their caregivers through the lens of the right to health. The right to health is an indispensable and fundamental human right that is recognized by numerous international instruments, including the WHO Constitution (1946), Article 25 of the Universal Declaration of Human Rights, and Article 12 of the International Covenant on Economic, Social, and Culture Rights (ICESCR) [21]. The core content of the right to health was further defined by General Comment No. 14, which is an official interpretation of Article 12 of the ICESCR. Accordingly, the right to health includes four core components: availability, accessibility, acceptability, and quality (AAAQ) [22]. Under each of these components, General Comment No. 14 offers detailed guidelines for UN member states to assess and improve health care for their citizens. In the literature, several studies have explored the feasibility of the AAAQ framework in identifying barriers to treatment [23–25]. In this study, we further elaborate the meaning of each component of AAAQ (Table 1) to reflect the topic of this study and guide the following activities, such as developing open-ended questions for our semistructured interviews.

**Table 1:** The AAAQ Framework with Refined Definitions

Domains	Definitions
Availability	From diagnosis to treatment, ensure functioning health services (e.g., clinical testing and treatment options) in sufficient quantity at all levels for patients with haemophilia A
Accessibility	Health facilities, services (e.g., infusion therapy), and medications (e.g., FVIII concentrate) must be accessible to all patients with haemophilia A. (1) Reach the most vulnerable patients (2) Physical accessibility: within safe physical reach (3) Financial accessibility: affordable to all (4) Sufficient educational information (e.g., disease recognition and management, self-infusion)
Acceptability	Health services (e.g., infusion therapy) need to be provided in a culturally sensitive way. Treatment options (e.g., episodic or prophylaxis) must be explained in an understandable way.
Quality	Medications (e.g., plasma-derived or recombinant FVIII) and techniques (e.g., infusion devices) are scientifically and medically appropriate and of good quality.

## Methods

### Study design

Inspired by the pursuit of the “right to health,” we used a qualitative approach [26] to explore barriers experienced or perceived by patients with haemophilia A who have initiated prophylactic treatment. After constructing open-ended questions based on the AAAQ framework, we used two qualitative interviewing methods to gather data: semi-structured personal interviews and focus groups. This ensured the collection of in-depth information. To safeguard quality control, we collected all the data face-to-face. We conducted personal interviews (in March of 2021) in a private room of the Shandong Haemophilia Treatment Centre (SHTC), and held focus groups (in July 2021) in meeting rooms of the Jinan University and Liaochen Hotels. We analysed the data using the thematic analysis approach in line with the method proposed by Braun and Clarke [27].

We took Shandong Province as the study area because it had the greatest number of patients with haemophilia A (2256 out of 17,779) in China [28]. Our research team collaborated with the SHTC, which is one of the six core centres of the China Haemophilia Treatment Centre Collaboration Network of China. The SHTC was established in 2001 under the supervision of the Health Commission of Shandong Province [29, 30]. Belonging to the National Haemophilia Registry System, the SHTC established a provincial haemophilia registry system to collect disease-related information from patients with haemophilia and other rare bleeding disorders in 2009. As of 2017, a total of 2586 haemophilia patients had participated in the registry. From recruiting patients known to be diagnoses with haemophilia A to collecting data, the SHTC has provided comprehensive and consistent support for this study.

### Recruitment

To obtain rich information from limited candidates, we used the purposive sampling technique to recruit participants. To protect patient privacy, we invited doctors to approach potential participants on the list and scheduled a time for personal interviews or focus groups if the potential participants were interested in this study. Supported by the SHTC, we were able to reach available candidates diagnosed with haemophilia A who met the inclusion criteria which are outlined below. The eligibility criteria for this study included patients or caregivers who were (1) affected by haemophilia A; (2) receiving treatments for more than one year; (3) used or using prophylactic treatment; and (4) able to give consent and willing to participate. In addition to these criteria, we concentrated on selecting patients who could provide rich information (e.g., patients who are communicative in their treatments). Potential participants were excluded if they had haemophilia B.

There is no accurate formula to calculate the exact number of participants needed for data saturation in qualitative research. Different studies have reported achieving data saturation after conducting different numbers of interviews, such as 6 [31], 12 [32], or 17 [33]. In this study, we first interviewed 12 patients, which is a discussion of saturation can be found in previous literature [32, 34]. However, since we were not able to achieve data saturation with the information provided by these 12 interviewees, we continued to recruit patients while performing thematic analysis. After 19 interviews, we found that no new themes emerged from the thematic analysis. Therefore, we believed that we had reached data saturation. To ensure data saturation, we used another method (i.e., focus groups) to collect data because the data generated by focus groups are believed to be deeper and richer than those obtained from personal interviews [35]. As recommended by the literature [32, 36], we invited 6 participants to each of the focus groups. We conducted focus groups while continuing the thematic analysis. After the third focus group, no new themes could be extracted from the analysis. Therefore, we concluded that data saturation was reached and stopped recruiting. Ultimately, we conducted 19 personal interviews and 3 focus groups.

A total of 37 participants (i.e., patients with haemophilia A alone, together with their caregivers, or represented by their caregivers) participated in this study, of whom 18 participated in focus groups and 19 participated in personal interviews. We interviewed 11 minor patients accompanied by their caregivers. For those 11 minor patients, we counted 1 minor patient and his caregiver together as 1 participant. We also had 2 adult patients who were very ill and unable to be interviewed. Considering the most proper utilization of the limited available patients, we interviewed their caregivers instead. For those two special groups of participants, we took measures to ensure that the voices of patients could be heard. For instance, in the interviews with minor patients and their caregivers, we encourage minor patients to say something before turning to their caregivers. For all interviews that included caregivers, we used communication techniques to ensure that the information provided by caregivers was on behalf of patients rather than the experiences of the caregivers themselves.

### Data collection

Four interviewers (JCF, JLW, LT, and LLL) who were experienced in qualitative research conducted all interviews. All four were trained to conduct interviews, as well as to fully capture key concepts relating to haemophilia A and prophylactic treatment. JCF and WJL were primarily responsible for the personal interviews, while LT and LLL conducted the focus groups.

For personal interviews, the interviewers started by introducing the purpose of this project to the participants and helping them to fully understand their important role in this research. Before obtaining informed consent from the participants, the interviewers explained the confidentiality-related measures and informed them that they were free to quit at any time and at any stage of this study. Then, the interviewers collected general demographic information before recording the interviews. Each personal interview started with open-ended questions (i.e., the questions in Box 1) and expanded with more questions inspired by the interviews. The interviews required the collaboration of two people: one was responsible for communicating with the patient(s), while the other took real-time notes and supplemented questions.

In each of the 3 focus groups, an experienced researcher acted as a moderator, and the observer monitored the process, transcribed all comments, and noted key points discussed. Once the participants arrived, they were given a written informed consent form and asked to complete a short questionnaire regarding age, gender, education, and medication use. Then, a brief introduction to the project was provided. Topics or open-ended questions (e.g., question 5 in Box 1) were provided to initiate group discussion. At the end of each focus group, the moderator summarized the discussion and encouraged the participants to provide more input.

On average, each personal interview lasted about 36 min, while each focus group lasted about 122 min. To express our great thanks to our participants for their devoted time and effort, we provided each of the participants with a thank you note and a small gift (i.e., RMB 100- approximately 14 USD or €13.50) after they finished the personal interviews or focus groups. The whole process of data collection was performed in Mandarin and recorded with the permission of the participants using an encrypted dictaphone.

**Box 1:** Examples of open-ended questions for semi-structured interviews

1.Would you like to share something about haemophilia A? Your personal story or the story of your kid 2.From the diagnosis to treatment, have you ever felt exhausted because of haemophilia A? 3.What is the most impressive negative experience of you or your kid during the disease journey? 4.According to you, which part(s)of prophylaxis treatment need to be improved? 5.Please sort these phrases in order according to your treatment experience and your wish (a)health services in enough quantities (b)health services are of good quality (c)health services are affordable (d)health services within safe physical reach	

Note: questions in the “Box” were initially asked in Mandarin and translated into English for the convenience of the exhibition

### Data analysis

Using a Dictaphone, JCF, and ZYL transcribed all interviews verbatim, and they and SPL checked and proofread the resulting transcripts against the audio. Independently, JCF and ZYL read and re-read the transcripts, familiarizing themselves with the content and writing down initial ideas for coding. Data were coded inductively, wherein codes were allowed to emerge from the data. Researchers coded the data manually, using NVivo as a supplemental marking and statistical tool. JCF and ZYL conducted the initial coding independently, then compared and discussed their results in order to cluster the codes into potential themes. Researchers identified eleven preliminary themes and drew an initial theme map to visually demonstrate the cross-linkages between emerging themes. By eliminating some initial themes, merging others, and categorizing remaining clusters into themes and subthemes, we ended up with nine subthemes and three overarching themes, organized and named to accurately and concisely represent our coded data. Then we invited clinicians, experienced qualitative researchers, and patients to review the names of themes and subthemes to ensure that the names were concise and representative of participants’ statements. In this article, themes and subthemes are illustrated with supportive quotations from the participants. Results were translated into English for reporting. The research was conducted in adherence with the consolidated criteria for reporting qualitative research (COREQ) [37].

### Ethical approval

This study was screened and approved by the Ethics Committee of the Centre for Health Management and Policy Research of Shandong University (No. ECSHCMSDU20200601). All participants gave their informed consent by signing the consent form. All participants were free to quit at any time and at any stage of this research. All documents were kept private and confidential. All audio-recorded interviews were reviewed only by the transcriber of this study and the principal investigator. Sensitive personal information, such as patients’ identification numbers, was strictly protected at all stages of this study.

## Results

Although we involved several caregivers in this study, the primary aim of this study was to explore barriers to the prophylactic treatment of patients with haemophilia A. Thus, Table 2 presents (only) the patients’ demographic information. The patients included in this study had a range of ages, educational levels, infusion methods, and other characteristics, with the exception of gender; all of the patients in this study were male. This was not only because haemophilia has a sex-linked recessive inheritance pattern, which means female patients are very rare [38], but also because female patients from SHTC, where we recruited participants, were mostly carriers with a low probability of needing prophylactic treatment.

**Table 2:** Demographic information of patients

Characteristics	Patient
**Gender**	
Male	37(100.0)
Female	0(0.0)
**Age (years)**	
< 18	11(29.7)
18–35	14(37.8)
36–50	9(24.3)
> 50	3(8.1)
**Educational level**	
No school or Primary school	8(21.6)
Secondary school	13(35.1)
High school or technical secondary school	8(21.6)
University degree and above	8(21.6)
**Clotting factor level**	
< 1% (severe)	19(51.4)
1–5%(moderate)	13(35.1)
5 to < 40% (mild)	5(13.5)
**Resource of medications**	
Domestic medications	6(16.2)
Imported medications	28(75.7)
Mixed	3(8.1)
**Infusion methods**	
Self- infusion	15(40.5)
Infusion by a doctor or nurse	13(35.1)
Infusion by a caregiver	6(16.2)
Mixed	3(8.2)

By using thematic analysis, nine subthemes and three themes were generated from the data. The nine subthemes represented barriers to prophylactic treatment that were perceived or experienced by patients with haemophilia A. To better depict relationships within the data, we further clustered those nine subthemes into three themes (Table 3). A detailed description of each theme and subtheme is provided, followed by supportive quotations from participants.

**Table 3:** Themes and subthemes of barriers in patients with haemophilia A

Themes	Subthemes
Suboptimal primary health care	A) Lack of health services for early diagnosis B) Inadequate supportive services for prophylaxis treatment in primary care C) Difficult in obtain FVIII concentrate close to home D) Lack of a clarified role of primary care nurses as an acceptable care provider for infusion therapy
Inadequate financial support	E) Financial concerns associated with prophylactic treatment F) Financial wariness surrounding newly developed treatment techniques
A lack of patient-centred care	G) Insufficient information regarding self-infusion and disease management H) Safety concerns associated with different product types I) Poor user experience of domestic-produced infusion devices

### Theme 1: suboptimal primary health care

China has established a primary to secondary/tertiary care referral system for delivering health care [39]. Ideally, the patient’s first visit should occur at the primary care level, and the patient should be referred to secondary/tertiary care when necessary. However, this is not a strict referral system, and patients have the option to make their first visit at any one of the three levels [40]. In this study, four subthemes were related to the suboptimal quality of health care provided at the primary level in China.

#### Subtheme A: lack of health services for early diagnosis

Although precise prevalence data are not available, haemophilia was found to be one of the diseases with the highest number of registered cases (as of December 31, 2020) according to the Chinese National Rare Diseases Registry System (created in 2016) [41, 42]. Despite the relatively high prevalence of haemophilia in China, patients still experienced delayed diagnosis, which greatly influenced their following treatment preferences. For instance, if haemophilia is not diagnosed early, more time and clinical examinations are often required to confirm suspected patients. Some interviewees mentioned that diagnostic services were not available in their location when they first experienced symptoms.

*My parents told me that when I was 2 years old, I bled quite easily after an injury, even if it was just a small accident. The bleeding could not be easily stopped. Health services were poor and inadequate for timely diagnosis at that time. Therefore, it was not until after I was 10 that the SHTC diagnosed my disease. (P11, Personal interview)*

*The health services and facilities in our area were not that good, so it was hard to figure out the exact reason for the abnormal bleeding. The disease was not diagnosed until we went to Shandong Provincial Hospital. (P10, Personal interview)*

#### Subtheme B: inadequate supportive services for prophylactic treatment in primary care

Prophylactic treatment requires that patients with haemophilia A receive regular infusions of FVIII concentrate, or that they receive prior infusions before engagement in activities that carry a high risk of injury. Some of our interviewees expressed financial concerns and the time-consuming problem of being infused at tertiary medical institutions.

*It is inconvenient to go to a medical institution for infusion because it is costly and time-consuming. (P2, Focus group)*

Since primary care clinics were perceived as to be more close-knitted with the local community, they were expected to make basic health care (e.g., infusion therapy) available for patients. However, it seems that primary care in China does not provide adequate support for patients with haemophilia A to adhere to prophylactic treatment, especially in terms of providing infusion therapy. Our interviewees expressed the difficulties of receiving infusion therapy at primary care clinics.

*It is hard to get infusion therapy from primary care clinics. Due to safety concerns or low profits, nurses at primary care clines are reluctant to provide intravenous therapy to you if the medication is not from the clinics where they are working. (P7, Focus group)*

Furthermore, deficiencies resulting in the missing role of primary care in supporting patients with haemophilia A might be exaggerated by the COVID-19 pandemic. One of our interviewees addressed her feelings of helplessness:

*I think it is not safe to go to hospitals (medical institutions at the secondary or territory level) during the pandemic. Therefore, I have to learn and practice infusion therapy for my kid at home. (P5, Personal interview)*

#### Subtheme C: difficulties in obtaining FVIII concentrate close to home

In China, FVIII concentrate is usually only available in certain general medical institutions of developed cities [43]. Patients with haemophilia A may have to travel a considerable distance from their homes to places where they can get medication if they live in rural areas where medical institutions do not offer a consistent supply of FVIII concentrates. They complained about the traveling distance and the time and effort it took to travel.

*Medical institutions in Dezhou City do not have FVIII concentrate. Therefore, I must travel to Jinan to get medication every time. Since I live in the rural area of Dezhou City, it takes me 5 h on average to go there and back. It is such an inconvenience! (P1, Personal interview)*

*I live in Linyi City, where no medical institution offers FVIII concentrate. It takes me almost a day on average each time to obtain FVIII concentrate. (P10, Personal interview)*

*It’s not that convenient for us to get FVIII concentrate. Every time, we need to go to the SHTC for medication. It still takes more than 3 h for us even with a high-speed train. We wish we could pick up medications in medical institutions where we live. (P10, Focus group)*

Furthermore, some of our interviewees who usually obtained their medication at general hospitals rather than from the SHTC reported the repeated process they had to go through each time to get FVIII concentrate.

*Before getting FVIII concentrate, you must do an outpatient register, wait to see a doctor, get a prescription, and pay for the fee generated from your visit and the medicine. Even worse, there is a separate queue for each stage, which is usually quite long. (P4, Focus group)*

As a consequence, some of our interviewees would prefer to get more medication during each visit than is currently allowed.

*I need to pick up medications once a month. 50 (doses) is the maximum number I am allowed to get each time. (P19, Personal interview)*

#### Subtheme D: lack of a clarified role of primary care nurses as acceptable care providers for infusion therapy

To protect patient safety, providing infusion services for patients with their self-carry medications is not allowed by most medical institutions according to their management regulations in China. Since the improper storage of FVIII concentrate (e.g., wrong temperature) may increase the risk of medical accidents, nurses working in primary care usually do not provide infusion therapy for patients with haemophilia A if the FVIII concentrate is from the patients’ stock. Exceptions exist in specific communities where people maintain strong relationships and demonstrate a high level of trust in their neighbours. In such a community, the influence of acquaintances would “force” nurses to provide infusion therapy even if this medical practice had a potential legal risk. Our interviewees reported their “lucky” or “unlucky” experiences in seeking infusion therapy from nurses working in primary care.

*I am familiar with doctors and nurses working in the clinic of our village. They are willing to provide infusion therapy for me. However, I often do not go there to avoid bothering them. (P6, Personal interview)*

*Usually, nurses do not provide infusion therapy because they are not allowed to. However, you may get the infusion if you are lucky enough to meet a “kind-hearted” nurse. The chances are pretty low. For example, I was refused by 6 clinics merely because the medication was from my stock. (P7, Focus group)*

### Theme 2: inadequate financial support

Adequate financial support helps to relieve patients’ stress associated with treatments [44]. In this study, two subthemes demonstrated a pattern of inadequate financial support for patients with haemophilia A in China.

#### Subtheme E: financial concerns associated with prophylactic treatment

Among the diverse problems and challenges of battling haemophilia A, the potential excessive financial burden of prophylactic treatment was very challenging. Some of our interviewees noted that they had to be careful about the overall expenses because the reimbursement rate would dramatically decrease if they exceeded their insurance scheme’s upper limit.

*The very first concern is the excessive expenses of prophylactic treatment. Infusing FVIII concentrate on a regular basis is very expensive for us. It takes over 3000 yuan per dose, and we need 5 to 6 doses on average each week. It costs at least 10,000 yuan to complete a course. We must consider this and manage no matter what kind of health insurance schemes we have registered. We must be careful not to exceed the upper limit of our insurance schemes. Otherwise, we must pay a large amount of money ourselves. (P17, Personal interview)*

*We have confidence in managing all kinds of difficulties except for reimbursement-related problems. If the total fee exceeds the limit of health insurance schemes, the cost would be on your own, which would be an unbearable burden to us. (P7, Focus group)*

Costly medication is another great barrier. Compared with efficacy and advantages, patients with haemophilia A tend to be more sensitive to the excessive financial burden associated with prophylactic treatment. To save money, some reduce the frequency of dose administration, forgo prescribed treatments, or even give up prophylactic treatment.

*There is a limit set by every kind of health insurance scheme on the total amount of reimbursement for a given patient with haemophilia A. The standard of this limit was framed in accordance with on-demand treatment rather than prophylactic treatment. Therefore, the reimbursed doses are far from adequate for an adult patient. To save money, I have to reduce the frequency of dose administration. It works fine and seems to make no difference. (P8, Personal interview)*

*I perform self-infusion at home. Still, it is costly. Sometimes I reduce the dosage of the FVIII concentrate or just skip one or two times of infusions. (P17, Personal interview)*

#### Subtheme F: Financial wariness surrounding newly developed treatment techniques

Patients with haemophilia A seemingly have a conservative attitude towards newly developed techniques. Since the coverage of newly developed treatment techniques has not yet been addressed by the basic medical insurance schemes in China, patients are less likely to switch to new products if the potential advantages are merely improvements in certain aspects, such as less preparation time and a lower risk of contamination.

*If the new technique only makes a small improvement, I prefer to keep the current way of treatment. (P4, Focus group)*

*Whether this drug can be reimbursed or not is my major concern. I need to make sure that the new drug can be covered by my insurance before switching. Otherwise, I would prefer to stay with my current way of treatment. (P10, Personal interview)*

However, not all kinds of newly developed therapies and techniques are unacceptable. Patients with haemophilia A show great interest in oral medications and highly advanced techniques, such as gene therapy.

*Hopefully, the treatment will change from intravenous to subcutaneous infusions and then from subcutaneous to oral medications. It would be great if gene therapy could be used widely. (P13, Personal interview)*

### Theme 3: a lack of patient-centred care

Patient-centred care addresses the special care needs of patients and encourages the active engagement of patients in managing their own health [45]. Putting patients at the heart of the care continuum requires that care-related information be shared fully and in a timely manner and emphasizes that care should focus on physical comfort and emotional well-being. In this study, three subthemes were related to patient-centred care.

#### Subtheme G: insufficient information regarding self-infusion and disease management

Patients with haemophilia A struggle with disease both mentally and physically. Since prophylactic treatment requires the regular infusion of FVIII concentrate, patients’ blood vessels may become increasingly fragile with the lengthened time of intravenous injection. However, it seems that supportive information regarding disease management is inadequate for most patients with haemophilia A, especially regarding how to manage the discomfort associated with self-infusion and how to prevent phlebitis and other complications caused by regular infusions.

*Self-infusion is difficult for us as patients. Less than half of us were willing to perform self-infusion, partially because we were not fully trained. We need special training programmes and supportive information for self-infusion. (P7, personal interview)*

#### Subtheme H: safety concerns associated with different product types

Plasma-derived and recombinant therapies are two major options for patients with haemophilia A. Although plasma-derived products were perceived as a more effective and much cheaper option, patients worried about the transmission of blood-borne viruses because the quality of the production process of plasma-derived concentrate had a history of being poor.

*I think the plasma-derived factor works fast and cheaper. However, it will increase the risk of infection. (P6, Focus group)*

Consequently, patients with haemophilia A prefer to choose recombinant products. However, recombinant products also have safety limits, particularly the risk of contamination during preparation. Most of those products are provided in the form of freeze-dried powder, which needs to be reconstituted before infusing.

*So, there is a possibility of contamination when the product is switched back and forth. Furthermore, you may waste a few miles during the process of preparing FVIII concentrate. (P15, Personal interview)*

#### Subtheme I: poor user experience of domestic-produced infusion devices

There is a difference in the basic package of the infusion device between domestically produced and imported devices. Patients who use a domestically produced infusion device usually need to buy a separate needle and normal saline. Although some domestically produced devices include needles, they are still not as good as imported devices according to patients’ user experiences.

*The needle included in the package of imported medication is of pretty good quality. It is a butterfly needle, which is smaller than the ordinary needle that we often use in the hospital. (P5, Personal interview)*

*Needles and normal saline are not included in the basic package of domestic-produced FVIII concentrate. You must buy them yourself. But the imported one provides all the supporting devices you need to infuse FVIII concentrate. (P12, Personal interview)*

Due to the potential risk of phlebitis and other complications resulting from long-term infusion, our interviewees expressed great hope for more user-friendly alternatives, such as subcutaneous infusion.

*I heard that subcutaneous infusion is comparatively convenient and simple and does not require training like intravenous infusion. (P2, Personal interview)*

## Discussion

In this study, we explored barriers to prophylactic treatment perceived or experienced by patients with haemophilia A in Shandong Province, China. The findings of this study indicated that patients with haemophilia A struggled with a wide range of intertwined barriers to prophylactic treatment. Based on the thematic analysis, we ultimately identified nine subthemes (barriers): (A) lack of health services for early diagnosis; (B) inadequate supportive services for prophylactic treatment in primary care; (C) difficulty in obtaining FVIII concentrate close to home; (D) lack of a clarified role of primary care nurses as acceptable care providers for infusion therapy; (E) financial concerns associated with prophylactic treatment; (F) financial wariness surrounding newly developed treatment techniques; (G) insufficient information regarding self-infusion and disease management; (H) Safety concerns associated with different product types; and (I) poor user experience of domestically produced infusion devices. Those intertwined barriers were further clustered into three groups reflecting institutional barriers that underlie perceived or experienced barriers: (1) poor primary health care (incl., A, B, C, and D); (2) inadequate financial support (incl., E and F); and (3) a lack of patient-centred care (incl., G, H, and I).

From diagnosis to treatment, primary health care is believed to be the best platform for meeting the needs of patients, especially for those who rely on regular health interventions to manage lifelong diseases [46]. However, in many places across China, particularly in less developed areas, primary health care is inadequate in terms of quality, reliability, and accountability [46]. In this study, the lack of facilities and well-trained health providers at the community level were highlighted by participants and accounted for most of their perceived barriers to prophylactic treatment. At the same time, the delivery system of certain medications (e.g., FVIII concentrate) does not include clinics at the primary care level, which limits the accessibility of potential drugs. Due to the suboptimal condition of primary health care, patients with haemophilia A were likely to be trapped in a vicious cycle of disease, in which patients who experienced delayed diagnosis might need extra clinical tests and more costly and time-consuming treatments, which decrease their treatment adherence and ultimately generate more severe complications.

Patients with rare diseases often experience diagnostic delays due to the high clinical complexity and low awareness of the disease [47]. As a rare disease, haemophilia A is unlikely to be noticed by patients until symptoms develop and become apparent. Therefore, delayed diagnosis is a relatively common experience for many patients with haemophilia A, often leading to extra clinical tests and unnecessary treatments that are costly and time-consuming and further decrease patients’ motivation for prophylactic treatment. These findings are consistent with those of published studies [28, 48, 49]. Due to the suboptimal primary health care in China, patients with haemophilia A bypass primary health care to seek treatment at the tertiary care level. However, this health care utilization pattern brings to the fore another vicious cycle caused by the fragmentation of health care delivery: poor primary health care, seeking care (e.g., diagnosis, subsequent diagnosis, collect FVIII, infusion) at tertiary care, limited access to infusion therapy at primary health care if medication is not from their stock, and low or non-adherence. In this study, most of our participants were influenced by this vicious cycle and complained about their difficulties in seeking infusion therapy and collecting FVIII concentrate close to home (identified as barriers A, B, and C).

Influenced by “acquaintance” factors, some participants broke this vicious cycle and received infusion therapy from nurses working at primary care clinics (identified as barrier D). In China, a distinctive feature of an acquaintance society is that people in the community see each other daily and help each other in their daily lives. Blood ties and local ties are the two most essential elements of acquaintance society [50]. In China, *mianzi* [51] (i.e., recognition by others of an individual’s social standing and position) and *renqing* [52] (i.e., one’s obligation of repaying favours or showing empathy to others in their social network) are two fundamental cultural characteristics that have substantial implications for understanding interpersonal dynamics. *Mianzi* and *renqing* can be used to explain why primary care nurses offer infusion services to patients with their self-carry medications. However, this treatment relationship is not solid, not only because providing infusion services as such makes nurses confront a high risk of litigation but also because patients face the problem of infusion again if one party in this relationship moves out of the acquaintance community.

Patients with haemophilia A often demonstrate a particular vulnerability, not only because of the suboptimal condition of primary health care but also because they do not receive adequate financial support (identified as barrier E). In China, patients’ medical expenditures can be partially reimbursed either through basic medical insurance or critical illness insurance (CII) [53]. Basic medical insurance has two basic schemes that target two different groups of people in terms of their employment status: urban employee basic medical insurance (UEBMI) for people who are employed and resident basic medical insurance (RBMI) for people who are unemployed or out of the labour force. The benefit amount of RBMI is lower than that of UEBMI, which contributes to the observed disparities in health care utilization. Furthermore, even within the same basic scheme, the benefit amount differs across municipalities in China [54], especially in terms of the reimbursement rate (ranging from 40 to 90%), the ceiling of each insurance scheme, and whether patients can claim reimbursements from basic medical insurance schemes and CII simultaneously. These differences greatly influence patients’ out-of-pocket medical expenses and thereby affect the acceptance and continuity of prophylactic treatment [55]. The second important demonstration of inadequate financial support is limited job opportunities. The high risk of severe functional impairment or disability could reduce patients’ opportunity to pursue higher education (reflected in Table 3) and high-paid jobs. As a possible consequence, patients with haemophilia A are less competitive in the labour market. Some of them are unemployed, and some of them receive temporary but low-paid jobs. Their employment realities and the low reimbursement rate of medical insurance together make prophylactic treatment unaffordable for some haemophilia A patients. The third important demonstration of inadequate financial support is associated with patients’ financial wariness of newly developed treatment techniques (identified as barrier F). Although newly developed techniques (e.g., subcutaneous infusion or new medications) may improve the treatment experience and may be applied with minimal training, haemophilia A patients are less likely to switch from their current treatment to newly developed ones. This is likely because most newly developed techniques are not yet covered by basic medical insurance. Thus, even if treatment experience improves, newly developed technologies may make a limited contribution to improving treatment adherence.

Patient-centred care and treatment adherence influence each other [56, 57]. In this study, the types of medications and methods of infusion were not patient-centred, which raised safety concerns among patients with haemophilia A and indirectly influenced their treatment adherence (identified as barrier H). The safety of medication use has long been the primary concern of patients with haemophilia A. In the time when plasma-derived FVIII was the only choice, the high risk of disease transmissions, such as AIDS and hepatitis B, made patients reluctant to take infusion therapy regularly and influenced their adherence. With the prevalence of recombinant FVIII, the risk of disease transmission decreased but was not eliminated due to the risk of contamination in the preparation process. In addition, the convenience of using medication is a primary safety-related concern of patients with haemophilia A. In our study, participants questioned the quality of domestically produced infusion devices, especially regarding convenience-related aspects of configuration (identified as barrier I).

Patient-centred care addresses the importance of empowering patients to be actively engaged in health care, such as by offering treatment-related educational and training programmes [45]. However, studies have reported that not only patients but also the public and health professionals perform poorly in rare disease awareness, which demonstrates the urgent need for educational information and training opportunities [47, 58, 59]. In this study, our data supported previous findings. The available opportunities were far from adequate to meet the demand of patients with haemophilia A in China (identified as barrier G). Studies have also indicated that self-care skills can be developed at a relatively young age through specialized physician instructions and individualized education [60]. Therefore, maximizing the accessibility of professional training programmes or individualized educational plans to patients with haemophilia A is essential and urgent, especially for minor patients. Providing educational and training opportunities is merely one aspect of putting patients at the heart of the care continuum. Lessons can be learned from other nations where integrated care for patients with haemophilia has been established to meet the physical, psychological, and emotional needs of these patients [61].

### Strengths and limitations

In this study, purposive sampling was used to include patients with differing ages, years of illness, and levels of education. We mainly employed qualitative research methods to understand patients’ experiences of prophylactic treatment since these methods allowed respondents to freely disclose their experiences, thoughts, and feelings without restrictions, providing a unique depth of understanding. Furthermore, this study explored the opinions and lived experiences of a unique population in a region where rare diseases, particularly their social and systemic aspects, have not yet been studied. There are also some limitations of this study. First, using only qualitative methods and purposive sampling implies that the study results cannot be extrapolated. The thoughts of certain patients may not have been represented due to the influence of “group effect” and other potential limitations of data collection methods. Second, the opinions of physicians and medical institutions were not included. The results may be biased due to reliance on the self-reports of patients. Third, the minors’ responses were provided with the assistance of their primary caregivers, and it is possible that the voices of young/underage patients are not adequately considered. Moreover, limited by recruiting patients from a single resource and the sex-linked inheritance of haemophilia, female patients with haemophilia A have received limited attention in this study.

## Conclusion

This study explored the opinions and lived experiences of patients with haemophilia A in using prophylactic treatment. Our findings not only provide an in-depth understanding of barriers encountered by Chinese patients with haemophilia A regarding prophylactic treatment but also address the urgent need to strengthen primary care, provide adequate financial support, and establish patient-centred care for these patients. The findings in this study may also serve as a useful reference for other nations when actions are needed to encourage early diagnosis and treatment of other rare diseases.

## Data Availability

All data relevant for the results are presented within the manuscript. Interview transcripts are not publicly available due to the sensitive nature of the data.
